# Genetic diversity and divergence among Korean cattle breeds assessed using a BovineHD single-nucleotide polymorphism chip

**DOI:** 10.5713/ajas.17.0419

**Published:** 2018-07-26

**Authors:** Seungchang Kim, Hyun Sub Cheong, Hyoung Doo Shin, Sung-Soo Lee, Hee-Jong Roh, Da-Yeon Jeon, Chang-Yeon Cho

**Affiliations:** 1 Animal Genetic Resources Center, National Institute of Animal Science, RDA, Namwon 55717, Korea; 2 Department of Genetic Epidemiology, SNP Genetics, Inc., Seoul 04107, Korea; 3 Department of Life Science, Sogang University, Seoul 04107, Korea

**Keywords:** BovineHD Chip, Heterozygosity, Linkage Disequilibrium (LD), Selection Signature, Effective Population Size

## Abstract

**Objective:**

In Korea, there are three main cattle breeds, which are distinguished by coat color: Brown Hanwoo (BH), Brindle Hanwoo (BRH), and Jeju Black (JB). In this study, we sought to compare the genetic diversity and divergence among there Korean cattle breeds using a BovineHD chip genotyping array.

**Methods:**

Sample data were collected from 168 cattle in three populations of BH (48 cattle), BRH (96 cattle), and JB (24 cattle). The single-nucleotide polymorphism (SNP) genotyping was performed using the Illumina BovineHD SNP 777K Bead chip.

**Results:**

Heterozygosity, used as a measure of within-breed genetic diversity, was higher in BH (0.293) and BRH (0.296) than in JB (0.266). Linkage disequilibrium decay was more rapid in BH and BRH than in JB, reaching an average r^2^ value of 0.2 before 26 kb in BH and BRH, whereas the corresponding value was reached before 32 kb in JB. Intra-population, inter-population, and Fst analyses were used to identify candidate signatures of positive selection in the genome of a domestic Korean cattle population and 48, 11, and 11 loci were detected in the genomic region of the BRH breed, respectively. A Neighbor-Joining phylogenetic tree showed two main groups: a group comprising BH and BRH on one side and a group containing JB on the other. The runs of homozygosity analysis between Korean breeds indicated that the BRH and JB breeds have high inbreeding within breeds compared with BH. An analysis of differentiation based on a high-density SNP chip showed differences between Korean cattle breeds and the closeness of breeds corresponding to the geographic regions where they are evolving.

**Conclusion:**

Our results indicate that although the Korean cattle breeds have common features, they also show reliable breed diversity.

## INTRODUCTION

Recent developments in genomics technology have enabled the analysis of genome-wide genetic structures [1]. The availability of single-nucleotide polymorphism (SNP) chips for massive genotyping has proven to be useful in genetically characterizing populations of animals and in assessing their degree of divergence [2]. Using these chips, it has become possible to study parameters such as linkage disequilibrium (LD) patterns, genetic diversity, and genome-wide association. In this regard, whole-genome SNP arrays are becoming widely used to study genetic diversity and are now considered among the tools routinely applied in animal breeding [3–5]. Studies of genetic relationships between cattle breeds provide useful information on livestock breeding programs and useful gene resource maintaining [6–8]. Breeds with a unique evolutionary history could potentially have a value not only in the maintenance of genetic diversity at the species level [9], but also for selection strategies aimed at genetic improvement through the application of genetics technologies [10].

In Korea, there are three main cattle breeds, which are distinguished by coat color: Brown Hanwoo (BH), Brindle Hanwoo (BRH), and Jeju Black (JB) [11]. The BH breed was established by a breeding and selection program as a major breeding stock. In Korea, there are over a million BH, whereas population sizes of the other two breeds number only a few thousand. Through strong artificial selection, the BH breed has become mainly specialized for meat traits [12]. Since the 1930s, when the Hanwoo breeding program began, the BH breed has been intensively selected based on carcass traits, such as carcass weight, eye muscle area, and intramuscular fat, through progeny tests. Hanwoo progeny tests have facilitated annual genetic gains in carcass traits that have increased dramatically in BH populations, and may have affected genomic regions associated with these carcass traits in this breed [13]. Understanding the genetic mechanisms that lead to phenotypic changes requires identification of the genomic regions that have been under long-term artificial selection. Strong artificial selection increases the frequency of favorable alleles at the loci that affect meat quality traits in meat production breeds. In this process, a small region of the genome surrounding mutations is also selected, resulting in a small genomic region that shows reduced variation [14]. This region of reduced variation is referred to as a selection signature, which is identified by nucleotide distributions around favorable mutations that differ statistically from those expected purely by chance [15]. Such selection signatures have revealed many genes that have been important in cattle selection.

The aim of the present study was to compare the genetic diversity and divergence of Korean cattle breeds using a BovineHD chip. Understanding the diversity in Korean cattle populations will enable researchers to develop better strategies for the breeding and conservation of these cattle.

## MATERIALS AND METHODS

### Animals and genotype assays

Sample data were collected from 168 cattle in three populations of BH (48 cattle), BRH (96 cattle), and JB (24 cattle). Blood of BH was collected by Animal Genetic Resources Research Center of National Institute of Animal Science. Blood of BRH was sampled from two local institutes (Gangwon Provincial Livestock Research Center and Chungbuk Veterinary Service Center). And, blood of JB was collected from Jeju special Self-Governing Provincial Livestock Institute. Blood samples of these three breeds were randomly collected, while avoiding parent-offspring or sib pairs where possible according to pedigree information of each institute. Genomic DNA for genotyping assays was extracted from a blood sample. The quantity and quality of the extracted DNA were evaluated using a spectrophotometer (NanoDrop 1000; Thermo Scientific, Waltham, MA, USA). The SNP genotyping was performed using the Illumina BovineHD SNP 777K Bead chip (Illumina, San Diego, CA, USA) [16]. This protocol was approved by the Committee on the Ethics of Animal Experiments of the National Institute of Animal Science.

### Quality control of the genotypes

The initial analysis of the images and the genotypes were carried out using GenomeStudio V2011.1 software (Illumina, USA). We excluded 3,290 SNPs because their genomic position was unknown and genotype clusters were not separated clearly in intensity data plotting. The SNPs with a call rate of less than 98%, and SNPs on the X chromosomes were also excluded from the analysis. A total of 735,182 SNPs were included in further statistical analysis. For selection signature discovery, autosome SNPs (n = 570,371) with a minor allele frequency (MAF) ≥0.05 were filtered out from the initial dataset. The LD coefficients (r^2^) of all pairs of SNPs were calculated using SVS8 software (Golden Helix SNP and Variation Suite; Bozeman, MT, USA), and then we excluded SNPs with an r^2^ value >0.5. A total of 226,694 SNPs were used for LD decay analysis. The final number of SNPs and individuals included in three Korean cattle breeds is shown in Table 1.

### Selection signature analysis

The cross population extended haplotype homozygosity (Rsb) analyses were conducted among BH, BRH, and JB using the *rehh* package [17] for R software. Candidate SNPs were defined as passing the Bonferroni correction threshold of p-value 8.77×10^−8^. Integrated haplotype score (iHS) analyses were conducted on BH, BRH, and JB respectively. This statistic was calculated for SNPs that passed the quality control criteria and exhibited a MAF of at least 0.05, since the algorithm of iHS has a limited power to calculate the statistic for fixed SNPs. Candidate regions were defined as in Rsb. As a prerequisite to the Rsb and iHS analyses, Beagle 3.3 [18] was used to phase the genotyped SNPs into the corresponding haplotypes. The fixation index (Fst) was estimated on the basis of the Wright F statistic [19] with use of SVS8. Candidate SNPs were defined as passing the Fst threshold 0.03.

### Population structure and genetic diversity analysis

The Fst were analyzed for the estimation of genetic distance and genomic relationship between samples using SVS8 software. The neighbor program of PHYLIP (phylogeny inference package) Version 3.695 (http://evolution.genetics.washington.edu/phylip.html) was used to construct a phylogenetic tree from Fst values.

### Linkage disequilibrium analysis

In the present study, we used the squared correlation coefficient between two loci (r^2^) as a measure of LD. This value denotes the ability of alleles at one marker to predict the alleles at a second marker [20,21]. For pairwise comparisons of all SNPs separated by a maximum distance of 100 Kb, the r^2^ value based on haplotype frequencies estimated via the expectation-maximization algorithm was predicted using SVS8 software. Subsequently, we analyzed the decay of LD between SNP pairs. For the purpose of graphical display, the average r^2^ values of each 2-kb distance were plotted. Effective population size (Ne) of each breed was estimated through SNP-based LD analysis with SNeP [22].

### Runs of homozygosity

Runs of homozygosity (ROH) were defined in each of BH, BRH, and JB using a sliding window approach of 50 SNPs in SVS8, as previously described for cattle by Purfield et al [23]. The ROH were estimated for each individual separately. Each ROH was categorized based on their physical length into 0.5 to 1 Mb, 1 to <5 Mb, 5 to <10 Mb, 10 to <15 Mb, 15 to <20 Mb and 20 Mb. For each of the aforementioned ROH length categories, the mean sum of ROH per population was calculated by summing all ROH per animal in that category and averaging this per breed population.

## RESULTS

### Genetic diversity

Using the Illumina BovineHD SNP 777K Bead chip, 99.4% of markers were genotyped in 95% of the samples in the three breeds, which indicates the suitability of the chip for genotyping the breeds studied (Table 1). The BH and BRH were calculated using 24 randomly selected samples to match JB samples. The proportion of SNPs with MAF >0.05 was 75.48% in BH, 76.16% in BRH, and 72.7% in JB, indicating that in all three breeds most SNP are segregating. The mean number of alleles was similar among breeds: 1.85 in BH and BRH, and 1.83 in JB. Overall, ~98% of SNPs were in Hardy–Weinberg equilibrium (p>0.01). Heterozygosity in the JB breed (0.266) was lower than that in both the BH (0.282) and BRH (0.284) breed (Table 1).

**Figure 1:**
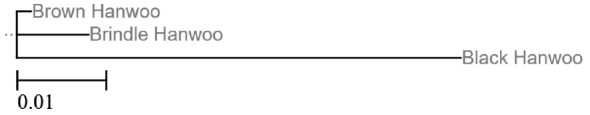
Neighbor-joining phylogenetic tree inferred using Fst values for the three cattle breeds. Scale bar indicates the distance (Fst value). The phylogenetic tree showed two main groups.

A phylogenetic tree was constructed from combining estimates of Fst values across overall SNPs (Figure 1). The tree shows two main groups. One of these groups includes the BH and BRH breeds, which are both raised on the Korean mainland. The other group comprises the JB breed, which is farmed on Jeju Island. This tree reveals a significant correlation between genetic and geographical distances. Our results are consistent with the classification of Korean cattle based on various morphological features proposed by Han et al [24].

### Selection signatures

We conducted a haplotype-based iHS analysis and Rsb analysis for selection signatures analysis. There were no significant loci in the genomic region for iHS analysis of BH and JB. Therefore, in order to clarify the genetic characteristics of BRH, we analyzed the selection signatures using Fst of single SNPs. The SNP criteria showing significant difference between two populations were considered to be a significant SNP when it had a Bonferroni correction p-value threshold (8.77×10^−8^) of iHS and Rsb analyses, and Fst threshold level is ≥0.25. A total of 184, 86 and 403 autosomal loci were potentially subjected to selection in BRH. Among these, 48, 11, and 11 loci were located in genomic regions. The list of genes is summarized in Table 2. There were many significant iHS across a broad range of chromosomes: four on BTA2, one on BTA4, three on BTA5, two on BTA6, four on BTA7, three on BTA8, four on BTA10, one on BTA11, two on BTA12, three on BTA13, one on BTA18, four on BTA19, two on BTA20, 11 on BTA22, and three on BTA22. Furthermore, the Rsb were discovered at significant loci on five chromosomes (one on BTA10, three on BTA19, one on BTA23, one on BTA26, and three on BTA28). Fst analysis identified significant loci on one chromosome (11 on BTA18).

### Linkage disequilibrium decay

**Figure 2:**
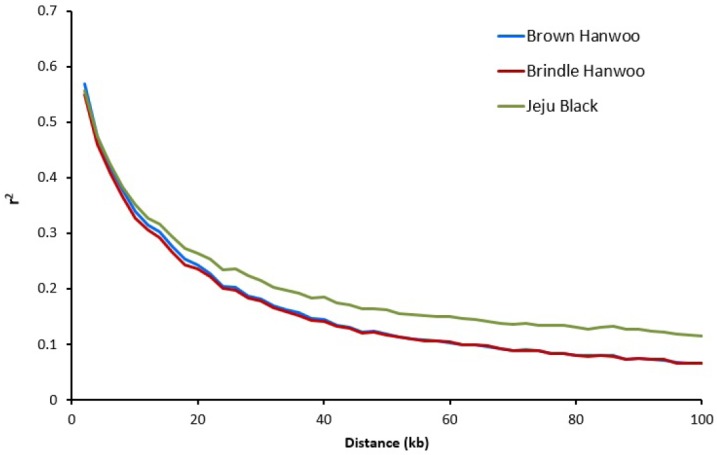
Average linkage disequilibrium (LD) decay (r^2^) from 0 to 100 kb for each of the three breeds included in the analysis. It used the squared correlation coefficient between two loci (r^2^). The LD decay was more rapid in Brown Hanwoo and Brindle Hanwoo than in Jeju Black.

The results of LD decay analysis up to 100 kb are shown in Figure 2. The analysis of decay corresponding to the HD chip shows that for the three breeds, r^2^ levels start at 0.568, 0.551, and 0.557 for BH, BRH, and JB, respectively, when using the 2 kb bin of SNPs. The decay of LD was more rapid in BH and BRH than in JB, reaching an average r^2^ value of 0.2 before 26 kb in BH and BRH, whereas the corresponding value was reached at 32 kb in JB. After these declines, the decay in each breed was considerably less steep, and at a distance of 100 kb, BH, and BRH reached an r^2^ value of 0.067 and JB reached an r^2^ value of 0.116.

### Effective population size

**Figure 3:**
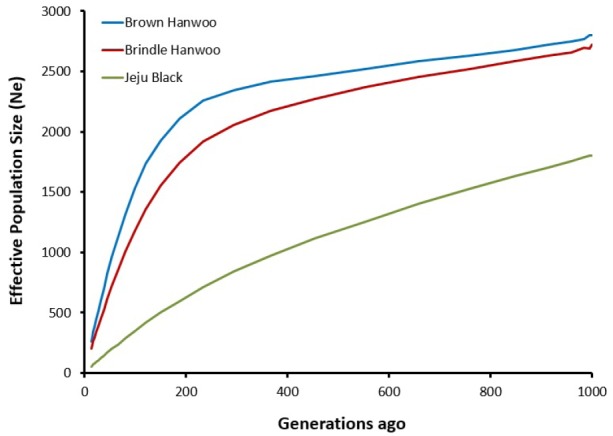
Effective population size (Ne) in the three breeds. Ne of each breed was estimated through single-nucleotide polymorphism-based linkage disequilibrium analysis with SNeP software. A decreasing trend in effective population size was observed.

Effective population size is strongly associated with genetic variability and adaptation. To understand the size and structure of these cattle populations, effective population size was calculated using LD estimates (r^2^). The three cattle breeds showed a declining trend in their effective population size (Figure 3). Among the breeds, BH and BRH had a high Ne of 260 and 202, respectively, until 13 generations ago, whereas JB had the lowest Ne of 55. This is, however, considerably higher than that reported by Sharma et al [25], who reported the Ne in BH and BRH to be 83 and 59 until 13 generations ago, whereas the Ne of JB was 67. The trends in LD and Ne are the same as those reported by Sharma et al [25]; however, the difference in estimated number could be attributed to differences in the number of samples used in the studies and the number of SNPs (50K vs 500K).

### Runs of homozygosity

**Figure 4:**
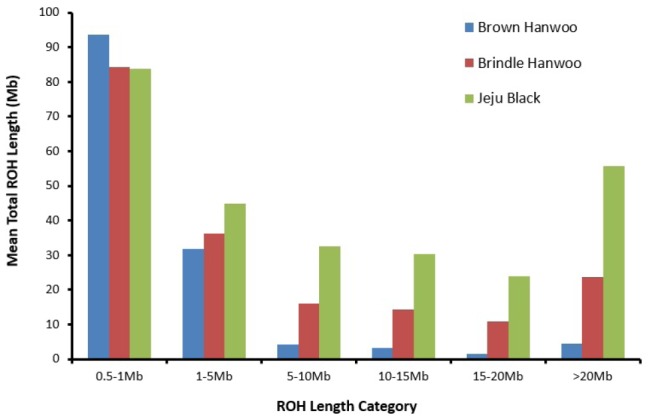
The mean sum of runs of homozygosity (ROH) per animal within each ROH length category using a sliding window approach of 50 single-nucleotide polymorphisms. Brindle Hanwoo and Jeju Black breeds have high inbreeding within breeds compared with Brown Hanwoo.

The ROH have different lengths and frequency depending on breeds. ROH were identified in all animals. Analysis of the distribution of ROH according to their size (Figure 4) showed that, for BH, ROH shorter than 5 Mb predominated. In contrast, a bimodal distribution with extremely long (>20 Mb) and relatively short (<5 Mb) ROH was found in the BRH and JB breeds. The mean ROH length was 23.1 Mb in BH, 30.8 Mb in BRH, and 45.2 Mb in JB. The BH breed had a greater mean proportion of autosomes, 93.7 Mb covered in a shorter ROH (<1 Mb), in comparison with the other breeds. Furthermore, the majority of the detected ROH were less than 5 Mb in length, with relatively few long ROH >20 Mb detected within the BH breed (mean ROH length for ROH >20 Mb was 4.4 Mb). The BH breed showed a tendency toward fewer ROH, whereas those of the BRH and JB breeds rose again in the ROH >20 Mb category. The JB and BRH breeds showed a relatively high value for ROH >5 Mb compared with the BH breed (mean ROH length for ROH >20 Mb was 55.7 Mb).

## DISCUSSION

The BH breed has been subjected to long-term artificial selection as part of a national breeding program aimed at improving meat quality and quantity. The JB and BRH breeds have both been excluded from this national breeding program, and consequently quality traits in these breeds have not been artificially selected. This has resulted in decreases in the population sizes of these breeds due to a lack of interest within the cattle industry. The JB and BRH varieties show some level of breed divergence from the BH breed, although this is distinctly less than between other well-characterized cattle breeds. From a purely genetic perspective, there is limited value in managing these populations independently; however, given their high social value in Korea, a separate breeding program aimed at maximizing diversity and improving fitness is warranted [26]. The Illumina BovineHD chip was designed using a large taurine reference population, and the results of the present study are consistent with those previously reported by O’Brien [27]. In addition, the overall call rate of our 168 animals showed the high performance of genotyping (>99%), indicating the strategic SNP selection of the HD chip for Korean cattle analysis.

Evidence for positive selection was obtained by calculating the value of iHS for a population [28], and Rsb [29], and Fst [19] between populations. Ancestral alleles were derived from BovineHD genotypes determined in a previous study [30]. We used three different selection signature discovery tools (iHS, Rsb, and Fst) to increase the power of detecting genomic signatures of positive selection. However, we failed to find overlapped loci among iHS, Rsb, and Fst analysis. Moreover, there were no significant loci in the genomic regions in the iHS analysis of BH and JB, and Rsb and Fst analyses of cross population BH-JB and BRH-JB pairs. However, a total of 184, 86, and 403 autosomal loci are potentially subjected to selection in BRH. Among these 48, 11, and 11 loci were located in genomic regions. There were many significant iHS across a broad range of chromosomes. In contrast, specific loci on Chr 10, 18, 19, 23, 26, 27, and 28 were discovered by Rsb and Fst analyses. We can assume that many of these genes are involved in functions that are associated with phenotypic changes in BRH. For example, we identified genes in cattle quantitative trait loci regions for feed efficiency and feeding behavior traits (aquaporin 4 [31]), eating behavior trait (calcium dependent secretion activator [32]), osteochondrosis (polypeptide N-acetylgalactosaminyltransferase 13 [33]), skin color trait (leucyl and cystinyl aminopeptidase [34]), pigmentation trait (melanocortin 1 receptor [35]), muscle trait (oxidative stress responsive 1 [36]), and hematological trait (tripartite motif containing 26 [37]).

JB showed lower heterozygosity and slower LD decay than BH and BRH. These differences could be attributed to population history events, including selection pressure, effective population sizes, and admixture with wild-type ancestors [25]. In contrast, BH and BRH showed lower levels of r^2^ compared with JB, which might be a reflection of the larger recent effective population sizes and relatively lower levels of inbreeding in the former two breeds. The patterns of LD and Ne are considerably affected by population historic events. In this study, we observed a sharp decline in the effective population size of all the cattle breeds. The sharp decline was observed at ~50 generations ago. This was the time of the formation of the current breeds, when selection and development of breeding programs had just begun. In the genetic structure of modern-day cattle, LD and Ne reflect various historic events and extensive artificial and natural selection.

The sum of an individual’s ROH coverage was used to infer the inbreeding level of an individual [23] Consanguinity may be indicated from the presence of long ROH; the longer such segments are, the more likely that recent inbreeding occurred within a pedigree [38]. The JB breed shows the highest inbreeding and the BRH breed shows a higher degree of inbreeding compared with the BH breed. The low genetic diversity in the JB and BRH breeds would inflate homozygosity and estimates of autozygosity in these populations. ROH were also mapped using their genetic positions and the abundance of ROH in different length classes was used to qualitatively evaluate the historical demography of each of the breeds.

To our Knowledge this study is the first to investigate the genome of a BRH cattle population for signatures of positive selection using high density genome-wide SNPs. Here, we have shown that a coat color-related gene (MC1-R) region is one of the selection signatures. The BH and BRH breeds showed similar overall genetic characteristics. Historically, these two breeds have been crossed without distinction on the mainland for long periods and share many genes. In particular, when BRH cattle are crossed with each other, cattle of brown, brindle, and black color all appear in the F_1_ generation. The BRH breed is thus considered to be the prototype of Korean cattle [39]. They have their own characteristics, but share many features. Compared with the BH and BRH breeds, the JB breed, which has been maintained on Jeju Island isolated from the mainland, shows a certain degree of genetic difference. Seo et al [40] reported that JB cattle form a population separate from the BH, BRH, and Korean black breeds. In addition, Yoon et al [11] concluded that the JB breed is genetically more distantly related to the BH breed than to the BRH breed. According to the theory that the cattle of Northeast Asia moved inland after they became domesticated, it can be assumed that the BH, BRH, and Korean black breeds originated in Korea, and that some Korean black cattle were subsequently transported to Jeju Island, where they have evolved as a separate population [41].

An analysis of differentiation based on high-density SNP chip analysis showed the various differences between Korean cattle breeds and the closeness of breeds corresponding to the geographic region in which they are evolving. Further studies will be necessary in order to determine the association between these genetic variations and various phenotypic traits.

## Figures and Tables

**Table 1:** Genetic diversity within three Korean cattle breeds

Breed	No. samples	Marker genotyped in 95% of the samples (%)	Number of polymorphic markers1)	Markers with MAF>0.051) (%)	Mean number of alleles1)	Markers in HWE (%) (p>0.01) 1)	Observed heterozygosity (SD)1)
Brown Hanwoo	48	99.4	654,582	75.48	1.85	98.88	0.282 (0.182)
Brindle Hanwoo	96	99.4	659,600	76.16	1.85	98.68	0.284 (0.181)
Jeju Black	24	99.4	609,685	72.70	1.83	99.10	0.266 (0.183)

MAF, minor allele frequency; HWE, Hardy–Weinberg equilibrium; SD, standard deviation.

1) Calculation based on randomly selected 24 samples of Brown Hanwoo and Brindle Hanwoo.

**Table 2:** Candidate selection signatures in BRH obtained from genome-wide SNP analysis in three Korean cattle breeds

rs#	BTA	Position	GeneID	Value	p-value	Methods1)
rs133768410	2	17310348	*MGC139239*	5.71	1.1E-08	iHS
rs136606289	2	41998558	*GALNT13*	5.55	2.9E-08	iHS
rs109429220	2	46751065	*LYPD6*	5.51	3.7E-08	iHS
rs42400978	2	53245523	*ARHGAP15*	5.81	6.4E-09	iHS
rs110543441	4	77460068	*OGDH*	5.80	6.7E-09	iHS
rs43709143	5	64646157	*SLC17A8*	5.55	2.8E-08	iHS
rs132649255	5	65021434	*ANO4*	5.75	8.8E-09	iHS
rs135611476	5	65566934	*UTP20*	5.60	2.2E-08	iHS
rs42500948	6	96900946	*C4orf22*	5.40	6.5E-08	iHS
rs137494018	6	102872168	*MAPK10*	5.55	2.8E-08	iHS
rs109162226	7	69865477	*SGCD*	5.60	2.1E-08	iHS
rs43129125	7	74367220	*ATP10B*	5.53	3.1E-08	iHS
rs110606217	7	92393984	*MBLAC2*	5.66	1.5E-08	iHS
rs133240533	7	98876412	*LNPEP*	5.67	1.4E-08	iHS
rs133207591	8	101803833	*SVEP1*	5.62	1.9E-08	iHS
rs43576537	8	107367416	*PAPPA*	5.94	2.8E-09	iHS
rs29019859	8	112426080	*CNTRL*	5.96	2.5E-09	iHS
rs29026593	10	54537392	*NEDD4*	6.30	2.9E-10	Rsb
rs136684474	10	56267173	*UNC13C*	5.50	3.8E-08	iHS
rs43637084	10	70168665	*SLC35F4*	5.63	1.8E-08	iHS
rs110040283	10	77239052	*SPTB*	5.42	5.9E-08	iHS
rs43644105	10	81096899	*ACTN1*	5.60	2.2E-08	iHS
rs133082379	11	37504767	*EML6*	5.77	8.1E-09	iHS
rs134518946	12	48954111	*KLF12*	5.74	9.7E-09	iHS
rs43702054	12	54982565	*NDFIP2*	5.81	6.1E-09	iHS
rs133421541	13	19142912	*PARD3*	5.86	4.6E-09	iHS
rs137035050	13	20050038	*NRP1*	5.40	6.8E-08	iHS
rs41628263	13	37145268	*MKX*	5.47	4.4E-08	iHS
rs133940147	18	14370567	*ANKRD11*	0.31	-	Fst
rs135905680	18	14512117	*SPG7*	0.31	-	Fst
rs136825205	18	14620678	*CDK10*	0.29	-	Fst
rs136080674	18	14635894	*VPS9D1*	0.26	-	Fst
rs136032320	18	14651461	*ZNF276*	0.32	-	Fst
rs110494166	18	14678403	*FANCA*	0.46	-	Fst
rs135530868	18	14746515	*TCF25*	0.44	-	Fst
rs134499247	18	14760377	*MC1-R*	0.44	-	Fst
rs134697549	18	14783535	*DEF8*	0.45	-	Fst
rs134353733	18	14822319	*DBNDD1*	0.42	-	Fst
rs134777248	18	14837991	*GAS8*	0.45	-	Fst
rs110992941	18	56911576	*MYH14*	5.93	3.0E-09	iHS
rs134697162	19	12260224	*BCAS3*	5.44	5.3E-08	iHS
rs135696145	19	20017811	*LYRM9*	5.60	2.1E-08	iHS
rs136501883	19	22171727	*TUSC5*	5.38	7.6E-08	iHS
rs134894253	19	30230290	*MYHC-EMBRYONIC*	5.81	6.3E-09	iHS
rs137391775	19	62330716	*PRKAR1A*	5.85	4.8E-09	Rsb
rs110514303	19	62455237	*MGC159964*	5.87	4.4E-09	Rsb
rs42699274	19	63125174	*CEP112*	5.95	2.6E-09	Rsb
rs134090120	20	25504490	*NDUFS4*	5.43	5.7E-08	iHS
rs41958923	20	67969388	*ADAMTS16*	5.67	1.4E-08	iHS
rs109923642	22	5154690	*TGFBR2*	6.00	2.0E-09	iHS
rs41995876	22	11649096	*MYD88*	5.70	1.2E-08	iHS
rs41995842	22	11695319	*OXSR1*	5.76	8.6E-09	iHS
rs42002387	22	11768489	*SLC22A13*	5.80	6.5E-09	iHS
rs42000489	22	15346591	*SEC22C*	5.45	5.1E-08	iHS
rs41640857	22	15696637	*GTDC2*	5.47	4.6E-08	iHS
rs133373037	22	19323943	*GRM7*	5.77	8.0E-09	iHS
rs108990174	22	37747536	*C3orf49*	5.91	3.5E-09	iHS
rs110659486	22	39026908	*CADPS*	5.96	2.6E-09	iHS
rs42008596	22	39238249	*PTPRG*	5.90	3.7E-09	iHS
rs134422044	22	46906743	*CACNA2D3*	5.43	5.7E-08	iHS
rs133683877	23	28565821	*TRIM26*	5.52	3.4E-08	Rsb
rs42047398	24	30362703	*AQP4*	5.41	6.2E-08	iHS
rs133648402	24	34562142	*GATA6*	5.57	2.5E-08	iHS
rs135865261	24	40481765	*LAMA1*	5.51	3.6E-08	iHS
rs136017859	26	21660800	*C26H10ORF6*	6.11	9.8E-10	Rsb
rs109276863	26	21677036	*SEMA4G*	6.46	1.0E-10	Rsb
rs41586295	27	42253264	*UBE2E2*	5.94	2.9E-09	Rsb
rs29020432	28	29117568	*PLA2G12B*	6.15	7.5E-10	Rsb
rs110736939	28	33155536	*KCNMA1*	5.78	7.5E-09	Rsb
rs134941983	28	35362739	*ANXA11*	6.11	9.7E-10	Rsb

BRH, Brindle Hanwoo; SNP, single-nucleotide polymorphism; BTA, *Bos taurus*; Rsb, cross population extended haplotype homozygosity; iHS, integrated haplotype score; Fst, fixation index.

1) For selection signature analysis Rsb, iHS, and Fst was conducted. Markers of Rsb and iHS were passed genome-wide significance level (p-value = 8.77×10^−8^). The Fst was estimated on the basis of the Wright F statistic and passed the Fst threshold 0.03.

## References

[1] Bovine HapMap C, Gibbs RA, Taylor JF (2009). Genome-wide survey of SNP variation uncovers the genetic structure of cattle breeds. Science.

[2] Shin D, Lee C, Park K-D, Kim H, Cho K-H (2017). Genome-association analysis of Korean Holstein milk traits using genomic estimated breeding value. Asian-Australas J Anim Sci.

[3] Ben Jemaa S, Boussaha M, Ben Mehdi M, Lee JH, Lee S-H (2015). Genome-wide insights into population structure and genetic history of tunisian local cattle using the illumina bovinesnp50 beadchip. BMC Genomics.

[4] Aslam ML, Bastiaansen JW, Elferink MG (2012). Whole genome SNP discovery and analysis of genetic diversity in Turkey (*Meleagris gallopavo*). BMC Genomics.

[5] McKay SD, Schnabel RD, Murdoch BM (2008). An assessment of population structure in eight breeds of cattle using a whole genome SNP panel. BMC Genet.

[6] Cañón J, Alexandrino P, Bessa I (2001). Genetic diversity measures of local European beef cattle breeds for conservation purposes. Genet Sel Evol.

[7] Uzzaman MR, Edea Z, Bhuiyan MSA (2014). Genome-wide single nucleotide polymorphism analyses reveal genetic diversity and structure of wild and domestic cattle in Bangladesh. Asian-Australas J Aim Sci.

[8] Edea Z, Bhuiyan MSA, Dessie T (2015). Genome-wide genetic diversity, population structure and admixture analysis in African and Asian cattle breeds. Animal.

[9] Toro MA, Caballero A (2005). Characterization and conservation of genetic diversity in subdivided populations. Philos Trans R Soc B Biol Sci.

[10] Flint APF, Woolliams JA (2008). Precision animal breeding. Philos Trans R Soc B Biol Sci.

[11] Yoon DH, Park EW, Lee SH (2005). Assessment of genetic diversity and relationships between Korean cattle and other cattle breeds by microsatellite loci. J Anim Sci Technol.

[12] Yoon DH, Kwon YS, Lee KY (2008). Discrimination of Korean cattle (Hanwoo) using DNA markers derived from SNPs in bovine mitochondrial and SRY genes. Asian-Australas J Anim Sci.

[13] Lee SH, Kim HC, Cho YM (2011). Genomic information and its application in Hanwoo (Korean native cattle) Breeding program - a mini review. Ann Anim Resour Sci.

[14] Lim DJ, Choi BH, Cho YM (2016). Analysis of extended haplotype in Korean cattle (Hanwoo) population. BMB Rep.

[15] Kim Y, Stephan W (2002). Detecting a local signature of genetic hitchhiking along a recombining chromosome. Genetics.

[16] Bovine Genome S, Analysis C, Elsik CG (2009). The genome sequence of taurine cattle: a window to ruminant biology and evolution. Science.

[17] Gautier M, Vitalis R (2012). rehh: an R package to detect footprints of selection in genome-wide SNP data from haplotype structure. Bioinformatics.

[18] Browning Sharon R, Browning Brian L (2007). Rapid and accurate haplotype phasing and missing-data inference for whole-genome association studies by use of localized haplotype clustering. Am J Hum Genet.

[19] Weir BS, Cockerham CC (1984). Estimating F-statistics for the analysis of population structure. Evolution.

[20] Ke X, Hunt S, Tapper W (2004). The impact of SNP density on fine-scale patterns of linkage disequilibrium. Hum Mol Genet.

[21] Boyles AL, Scott WK, Martin ER (2005). Linkage disequilibrium inflates type i error rates in multipoint linkage analysis when parental genotypes are missing. Hum Hered.

[22] Barbato M, Orozco-terWengel P, Tapio M, Bruford MW (2015). SNeP: a tool to estimate trends in recent effective population size trajectories using genome-wide SNP data. Front Genet.

[23] Purfield DC, Berry DP, McParland S, Bradley DG (2012). Runs of homozygosity and population history in cattle. BMC Genet.

[24] Han TW, Kim SK, Jeon Y (1966). The classification and distribution of Korean cattle tick. Rural Res Report, RDA.

[25] Sharma A, Lim D, Chai HH, Choi BH, Cho Y (2016). Demographic trends in Korean native cattle explained using bovine SNP50 beadchip. Genomics Inform.

[26] Strucken EM, Lee SH, Jang GW, Porto-Neto LR, Gondro C (2015). Towards breed formation by island model divergence in Korean cattle. BMC Evol Biol.

[27] Pérez O’Brien AM, Mészáros G, Utsunomiya YT (2014). Linkage disequilibrium levels in *Bos indicus* and *Bos taurus* cattle using medium and high density SNP chip data and different minor allele frequency distributions. Livest Sci.

[28] Voight BF, Kudaravalli S, Wen X, Pritchard JK (2006). A map of recent positive selection in the human genome. PLoS Biol.

[29] Tang K, Thornton KR, Stoneking M (2007). A new approach for using genome scans to detect recent positive selection in the human genome. PLoS Biol.

[30] Matukumalli LK, Lawley CT, Schnabel RD (2009). Development and characterization of a high density SNP genotyping assay for cattle. PLoS One.

[31] Reyer H, Shirali M, Ponsuksili S (2017). Exploring the genetics of feed efficiency and feeding behaviour traits in a pig line highly selected for performance characteristics. Mol Genet Genomics.

[32] Do DN, Strathe AB, Ostersen T (2013). Genome-wide association study reveals genetic architecture of eating behavior in pigs and its implications for humans obesity by comparative mapping. PLoS ONE.

[33] Wittwer C, Hamann H, Distl O (2009). The candidate gene XIRP2 at a quantitative gene locus on equine chromosome 18 associated with osteochondrosis in fetlock and hock joints of south German coldblood horses. J Hered.

[34] Nie C, Zhang Z, Zheng J (2016). Genome-wide association study revealed genomic regions related to white/red earlobe color trait in the Rhode Island Red chickens. BMC Genet.

[35] Gutiérrez-Gil B, Wiener P, Williams JL (2007). Genetic effects on coat colour in cattle: dilution of eumelanin and phaeomelanin pigments in an F2-Backcross Charolais × Holstein population. BMC Genet.

[36] Ponsuksili S, Murani E, Trakooljul N, Schwerin M, Wimmers K (2014). Discovery of candidate genes for muscle traits based on GWAS supported by eQTL-analysis. Int J Biol Sci.

[37] Zhang F, Zhang Z, Yan X (2014). Genome-wide association studies for hematological traits in Chinese Sutai pigs. BMC Genet.

[38] Kirin M, McQuillan R, Franklin CS (2010). Genomic runs of homozygosity record population history and consanguinity. PLOS One.

[39] Lim DJ, Lee SH, Choi BH (2014). Identification of selection signals in Chikso (Brindle Hanwoo) using Rsb method. J Agric Life Sci.

[40] Seo K, Mohanty TR, Choi T, Hwang I (2007). Biology of epidermal and hair pigmentation in cattle: a mini-review. Vet Dermatol.

[41] Choi TJ, Lee SS, Cho KH (2015). Genetic diversity of traditional Korean cattle breeds based on microsatellite polymorphisms. J Agric Life Sci.

